# A network pharmacology‐based approach to explore the effects of Chaihu Shugan powder on a non‐alcoholic fatty liver rat model through nuclear receptors

**DOI:** 10.1111/jcmm.15166

**Published:** 2020-03-18

**Authors:** Huan Nie, Yuanjun Deng, Chuiyang Zheng, Maoxing Pan, Jiqian Xie, Yupei Zhang, Qinhe Yang

**Affiliations:** ^1^ School of Traditional Chinese Medicine Jinan University Guangzhou China

**Keywords:** Chaihu Shugan powder, network pharmacology, non‐alcoholic fatty liver disease, nuclear receptors, traditional Chinese medicine

## Abstract

The pathogenesis of non‐alcoholic fatty liver disease (NAFLD) is still not fully understood, and currently, no effective pharmacotherapy is available. Nuclear receptors (NRs) are important biological participants in NAFLD that exhibit great therapeutic potential. Chaihu Shugan powder (CSP) is a traditional Chinese medicine (TCM) formula that has a wide therapeutic spectrum including NAFLD, but the effective components and functional mechanisms of CSP are unclear. We adopted a network pharmacology approach using multiple databases for Gene Ontology (GO) enrichment analysis and the molecular complex detection (MCODE) method for a protein‐protein interaction (PPI) analysis, and we used molecular docking method to screen the NR targets and determine the corresponding CSP components. The screening results were validated through a NAFLD rat model that was used to explain the possible relationship between CSP and NAFLD. Finally, we screened PPARγ, FXR, PPARα, RARα and PPARδ as target genes and *quercetin*, *kaempferol*, *naringenin*, *isorhamnetin* and *nobiletin* as target compounds. The five components were detected through high‐performance liquid chromatography‐mass spectrometry (HPLC‐MS), the results of which aligned with the docking experiments of PPARγ, PPARα and PPARδ. After CSP intervention, the NAFLD rat model showed ameliorated effects in terms of bodyweight, hepatic histopathology, and serum and liver lipids, and the mRNA levels of PPARγ, FXR, PPARα and RARα were significantly changed. The results from this study indicate that CSP exhibits healing effects in an NAFLD model and that the network pharmacology approach to screening NR targets and determining the corresponding CSP components is a practical strategy for explaining the mechanism by which CSP ameliorates NAFLD.

## 1 INTRODUCTION

Non‐alcoholic fatty liver disease (NAFLD) is a metabolic stress injury to the liver that is closely related to insulin resistance and is based on genetic susceptibility without a history of excessive alcohol consumption. The NAFLD disease spectrum includes non‐alcoholic simple fatty liver (NAFL), non‐alcoholic steatohepatitis (NASH), and cirrhosis and hepatocellular carcinoma (HCC).^1^, ^2^ NAFLD has an estimated global prevalence of 24%, indicating that it is the most common cause of liver disease worldwide.^3^ Currently, there is no approved pharmacotherapy for NAFLD. Lifestyle modification, control of comorbid metabolic illness and some pharmacological options, such as vitamin E, pioglitazone and pentoxifylline, are the treatment options.^4^

Nuclear receptors (NRs) are ligand‐activated transcription factors, of which 48 members constitute the largest family of transcription regulatory mechanisms.^5^ In the progression of NAFLD, NRs are important biological participants involved in insulin resistance and fatty acid (FA) synthesis,triglyceride (TG), total cholesterol (TC) and glucose (GLU) metabolism; bile acid homeostasis; drug detoxification; inflammation; regeneration; fibrosis; tumour formation; etc.^6^, ^7^ Some NRs are sensitive to lipid metabolic processes which may play important roles in NAFLD.^7^ Therefore, the pathogenesis and pharmacological function of NRs in the mechanism of NAFLD deserve further research.

Chaihu Shugan powder (CSP) is a traditional Chinese medicine (TCM) formula consisting of seven herbs: *Radix Bupleuri*, *Citrus Reticulata*, *Chuanxiong Rhizoma*, *Paeoniae Radix Alba*, *Aurantii Fructus*, *Cyperi Rhizoma* and *Radix Glycyrrhizae*. CSP, first recorded in *Jingyue Complete Library* of the Ming Dynasty China, has been clinically used to treat digestive diseases and emotional diseases in China and other Far East countries for almost four centuries. Current studies have reported that CSP has therapeutic potential for the treatment of NAFLD because it ameliorates insulin resistance, lipid peroxidation, steatohepatitis, etc,^8^, ^9^, ^10^ but the chemical components and functional mechanisms of CSP have not been fully studied. Therefore, an exploration into the underlying mechanism of CSP in NAFLD is necessary.

The network pharmacology approach is a big data integration method based on a large number of database resources and statistical algorithms that are used to observe the synergy of multiple components, targets and mechanisms of disease and can be applied to broad‐perspective analyses of TCM drugs.^11^, ^12^, ^13^ As with other natural medicines, CSP contains abundant chemical components that indispensably correspond to many target genes and biological mechanisms.^14^ Therefore, it is very difficult to fully clarify the effective components and functional target range and specifically to elucidate the therapeutic mechanisms of CSP. Therefore, we adopted a network pharmacology approach to screen NR targets and corresponding components of CSP and conducted verification experiments on a rat model of NAFLD to further elucidate the relationship between CSP and NAFLD.

## 2 MATERIALS AND METHODS

### 2.1 Components and target gene screening of CSP

The TCMSP, BATMAN‐TCM, TCMID and TCMGeneDIT databases were used to identify the chemical components of CSP. DrugBank and PubChem were used to search for the information of chemical compounds and target genes of CSP. NCBI, GeneCards and DisGeNET were used to search for the therapeutic target genes of NAFLD. UniProt and Ensembl were used to convert protein names to gene names (ENSG identifiers). The target genes at the intersection of CSP and NAFLD (C&N) were regarded as potential target genes through which CSP influences NAFLD, and the corresponding chemical compounds of the C&N candidates were thought to be possible therapeutic components that affect NAFLD. Then, the NRs were screened out from the C&N candidates and regarded as potential therapeutic NR targets of CSP in NAFLD. Similarly, the CSP compounds corresponding to these NRs were regarded as possible therapeutic agents for NAFLD treatment.

### 2.2 High‐potential target screening of CSP

As there are differences in data sources, data storage and statistical algorithms, to identify high‐potential NR targets, we integrated multiple databases and conducted Gene Ontology (GO) enrichment analysis using a comprehensive approach, which included the biological process (BP), molecular function (MF) and cellular component (CC) categories. From the results of the BP, MF and CC analysis of each database, we selected the top thirty clusters based on statistical significance and then ranked the total NR frequency in all these clusters in descending order. Finally, the top five NRs were selected as the high‐potential target genes of CSP. For GO enrichment analysis, the smaller *P*‐value of a cluster was considered to indicate greater biological significance of CSP treatment of NAFLD; hence, the NRs included in these clusters were regarded as more effective potential therapeutic targets. In addition, we also integrated multiple databases to conduct a signalling pathway enrichment analysis of the C&N groups and then selected the top thirty pathways derived from each database based on statistical significance and determined the expression of the top 5 NRs in each pathway. Protein‐protein interaction (PPI) analysis of the C&N data was conducted by STRING, and the highly interconnected regions in the PPI network were found through the molecular complex detection algorithm (MCODE) plugin in Cytoscape with the parameters set to the default values. Cytoscape was also used to visualize the topological structure of the interaction network. Each node in the network represented a target or compound, and each line represented the connection of the target to the compound or the target to the target.

### 2.3 High‐potential component screening of CSP

As the top 5 NRs may function through more than one compound of CSP, it is necessary to conduct an ADME pharmacodynamic screen based on oral bioavailability (OB) > 30% and druglikeness (DL) > 0.18. After the compounds that corresponded to top 5 NRs were ranked by frequency, the top 5 compounds were selected as the final target CSP components. Similar to the top 5 NRs, the top 5 compounds could be considered high‐potential therapeutic components of CSP for treating NAFLD, presupposing that they function through the top 5 NRs.

### 2.4 Animals and treatments

Thirty male SPF Wistar rats weighing 200 (±20) g at the beginning of the feeding period were provided by Pengyue Laboratory Animal Center, Jinan, Shandong, China (Licence: SCXK (Lu) 20140007). The rats were raised in the SPF animal room of the Institute of Laboratory Animal Science, Jinan University, Guangzhou, Guangdong, China (Licence: SYXK (Yue) 2017‐0174). Normal chow (3.4 kcal/g, consisting of 13% kcal fat, 22% kcal protein and 65% kcal carbohydrate without cholesterol; LAD0011) and high‐fat chow (4.2 kcal/g, consisting of 37% kcal fat, 22% kcal protein and 41% kcal carbohydrate with 12 g cholesterol/kg; TP0800) were provided by Trophic Animal Feed Co., Ltd. Both chows contained saturated, monounsaturated and polyunsaturated FAs. The CSP crude herbs were processed into water‐soluble granules by Jiangyin Tianjiang Pharmaceutical Co., Ltd. (containing 9.6 g/kg crude herbs). The dosage proportion of *Radix Bupleur*i: *Citrus Reticulata*: *Chuanxiong Rhizoma*: *Paeoniae Radix Alba*: *Aurantii Fructus*: *Cyperi Rhizoma*: *Radix Glycyrrhizae* was 6:6:5:5:5:5:3. The CSP granules were dissolved in water (0.2 g/mL) at 80°C and cooled to room temperature before gavage (the CSP calories were calculated based on 4 kcal/g carbohydrate). The rats were isolated and fed for one week while they adapted to the environment and then randomly divided again into a normal control (NC) group (n = 10), high‐fat diet (HFD) control group (n = 10) and CSP intervention (CSP) group (n = 10) according to the random number table method. There were 3‐4 rats fed in one cage for a total of 8 weeks. The NC group rats were fed freely with normal chow, and the HFD and CSP group rats were fed freely with high‐fat chow. All the rats had free access to drinking water. In addition, the CSP group rats were given a weight‐corresponding dosage of CSP (10 mL/kg), and the NC and HFD group rats were given the same dosage of deionized water. The room temperature was 22 (±2)°C, the relative humidity was 55 (±5)%, light and dark conditions were alternated every 12 hours, and the rat cages were cleaned every 3 days.

### 2.5 Biochemical detection

After 8 weeks of feeding, the rats were fasted for 12 hours, but had free access to drinking water, and then were intraperitoneally anesthetized with pentobarbital sodium (40 mg/kg). After the rats were unconscious, abdominal aortic blood was collected and placed at room temperature for 30 minutes, and then, the serum was collected after centrifugation for 10 minutes (4°C, 1509.3*g*). Serum alanine aminotransferase (ALT), aspartate aminotransferase (AST), alkaline phosphatase (ALP), TC, TG, GLU, high‐density lipoprotein cholesterol (HDL‐c) and low‐density lipoprotein cholesterol (LDL‐c) were measured by an automatic biochemical analyzer (Chemray 240, Rayto Life and Analytical Sciences). Liver samples (100 mg) were cut into pieces with scissors and mixed in 0.9 mL absolute ethyl alcohol, homogenized with a high‐throughput tissue grinder and then centrifuged for 10 minutes (4°C, 1509.3*g*). Then, the supernatants were extracted. The TG and TC levels in the liver homogenate supernatants were measured with TG and TC kits (A110‐2‐1 and A111‐2‐1, respectively, Nanjing Jiancheng Bioengineering Institute) according to the manufacturer's protocols.

### 2.6 Pathological observation

Fresh liver samples were cut into 1.0 × 0.5 × 0.5 cm pieces and fixed in 10% neutral formaldehyde solution. After being dehydrated and embedded in paraffin, 4‐μm tissue sections were cut with a paraffin microtome in preparation for HE staining. Liver tissues (1.0 cm^3^) were fixed in OCT solution for 15 minutes, and then 8 μm tissues were cut on a freezing microtome in preparation for Oil red O staining. For the same samples, 1.0 mm^3^ liver tissues were cut into pieces and fixed in 2.5% glutaraldehyde electron microscopy fixative solution for 24 hours at 4°C; washed with PBS; fixed with 1% osmium acid fixative solution; dehydrated with acetone; embedded with epoxy resin, which was allowed to polymerize at 60°C for 24 hours; and then cut into ultrathin sections. The 40‐ to 50‐nm‐thick tissue sections were stained with uranium acetate and lead citrate. Liver lipid droplets were stained with 100×, 200× and 400× HE and 200× Oil red O and then observed under an optical microscope (DM2000, Leica), and ultrastructure of the liver tissue was observed under a transmission electron microscope (TEM) (Hitachi) at 7000×. ImageJ software was used to calculate the lipid area in 200× Oil red O‐stained sections.

### 2.7 Quantitative real‐time PCR

Total RNA was extracted from the liver tissue using TRIzol reagent (15596026, Thermo Fisher). Reverse transcription reactions were conducted according to the instructions for the PrimeScript reagent kit (RR047A, TaKaRa). qPCR amplification was conducted using a 25 μL reaction system with a PCR amplifier (iQ5, Bio‐Rad) according to the instructions of the qPCR reagent kit (RR820A, TaKaRa). The relative quantification of RNA, in number of folds, was calculated by the 2^−ΔΔCt^ method. The gene sequences were acquired from GenBank, and GAPDH was used as the internal reference. The primers were synthesized by Shanghai General Biotech Co., Ltd., (primer information is listed in Table S2).

### 2.8 Automatic Western blotting

The sample protein was extracted from liver tissues after the cells were lysed by RIPA buffer (89901, Thermo Scientific) at 4°C for 20 minutes and centrifuged for 10 minutes (4°C, 11180.0*g*). The secondary antibodies were anti‐PPARγ (ab209350, Abcam), anti‐PPARα (ab24509, Abcam), anti‐FXR (NBP2‐16550, Novus), anti‐RARα (ab254098, OriGene) and anti‐PPARδ (ab23673, Abcam). The protein was quantified completely by automatic Western blotting through a Wes automated system (ProteinSimple). The dynamic linear range of the standard protein was diluted in a gradient from 0.125 to 4 mg/mL. The Western blotting process was conducted according to the instructions for the Wes system, which differs from the traditional method. The machine was run with default parameters, and imaging and analysis were performed using Compass software (ProteinSimple).

### 2.9 HPLC‐MS

To detect the top 5 compounds in CSP, the CSP granules, which were from the same batch produced for rat feed, were precisely weighed according to the ratio described in the Animals and treatments section. The CSP granules were filtrated through a 0.25 μm microporous membrane, dissolved in 50 mL formaldehyde solution and oscillated for 5 minutes. Then, the CSP solution was ultrasonically processed for 30 minutes and diluted in a gradient from 0.1 to 1000 μg/mL. The standard drugs *quercetin* (Q111273‐20 mg, Aladdin, purity ≥ 98.5%), *isorhamnetin* (I109591‐20 mg, Aladdin, purity > 98%), *naringin* (N107346‐20 mg, Aladdin, purity ≥ 98%), *kaempferol* (Mole‐M4368, AbMole, purity > 98.0%) and *nobiletin* (N130078‐10 mg, Aladdin, purity > 95.0%) were diluted in a gradient from 0.1 to 1000 μg/mL. High‐performance liquid chromatography (HPLC) (1260, Agilent) with 4.6 × 250 mm and 2.7 μm chromatographic columns (Poroshell 120 EC‐C18, Agilent) was used. The mobile phase consisted of 2‰ formic acid water‐acetonitrile (96:4), the column temperature was 40°C, the flow rate was 450 μL/min, and the injection volume was 5 μL. Gas‐assisted electrospray ionization (ESI) was used for mass spectrometry (MS), and the detection method was based on multi‐ion reaction measurement (MRM).

### 2.10 Molecular docking

PubChem and PDB were used to find the chemical and conformational information of the relevant proteins and small‐molecule compounds. The keywords associated with the proteins were determined, and the candidates were selected according to the following criteria: a crystal resolution of <3 Å for protein structures obtained by the X‐ray crystal diffraction method, a well‐defined type of protein and a protein with small‐molecule ligands. The AutoTools software was used to remove the redundant protein chains, ligands and water molecules with hydrogenation before running docking experiments. The AutoGrid software was used to calculate the energy lattice points with the grid box coordinates of 20 × 20 × 20. AutoDock Vina was used to simulate the docking condition between proteins and small molecules. The Schrodinger software was used to analyse the preferential conformation and map the simulation (databases are listed in Table S1).

### 2.11 Statistical method

In addition to the built‐in statistical algorithms in the databases and/or software used for network pharmacology, all the statistical calculations of the one‐way analysis of variance (ANOVA) were performed using GraphPad Prism 8.0 software. The experimental results are presented as the means ± standard deviation, and *P* < .05 indicates significance.

## 3 RESULTS

### 3.1 CSP components and targets

**Figure 1:**
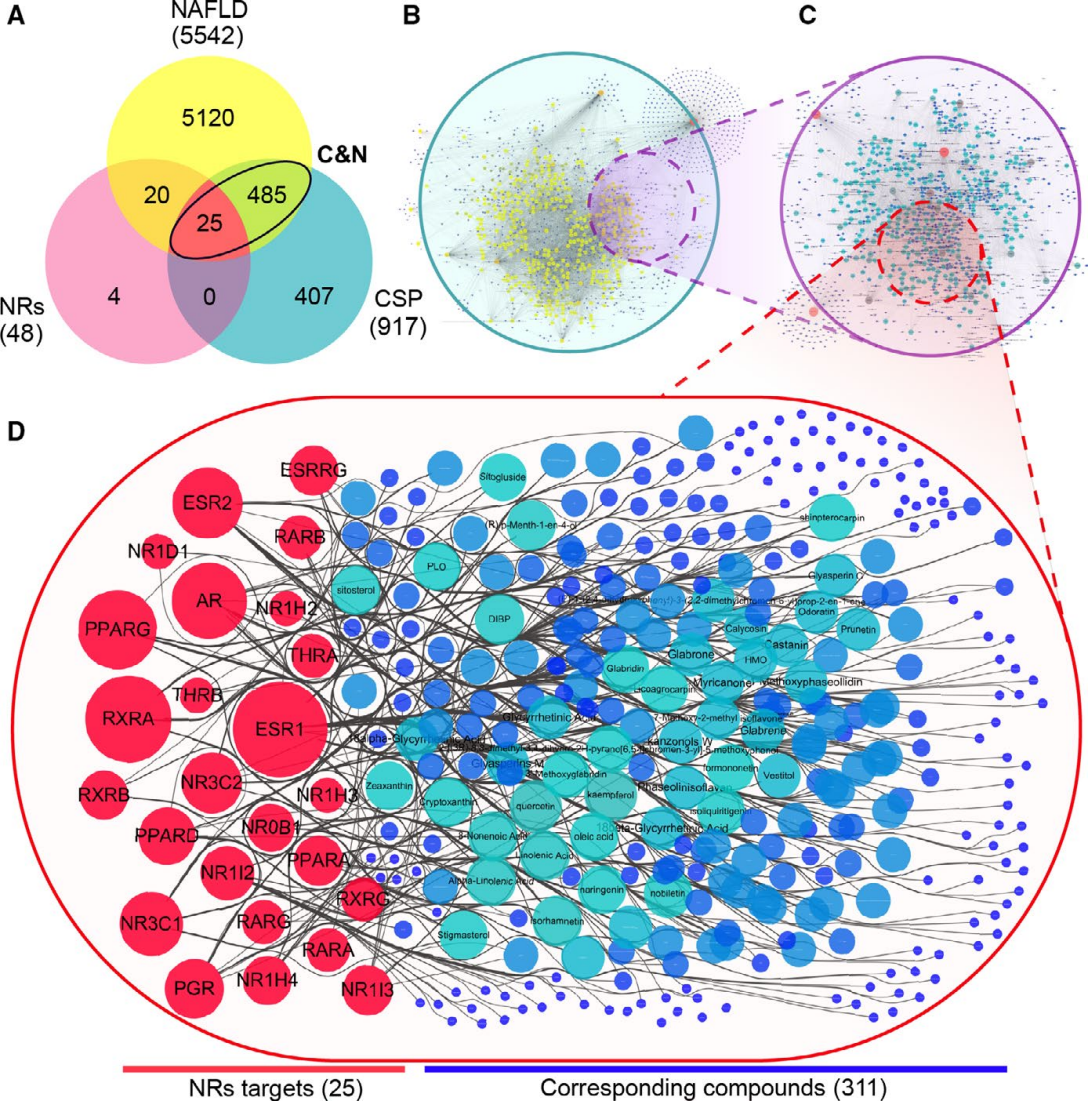
The general target‐compound relation of CSP, C&N and NRs. A, The quantity of intersecting NAFLD, CSP, NRs and C&N target genes. B, The compound‐target network of CSP. C, The target‐compound network based on the C&N intersection. D, The target‐compound network of the NRs in the C&N network. In networks B‐D, each edge represents the connection between the target node and compound node, and the size of the node represents the total number of connecting edges

After repeated names were removed, 730 compounds and 917 corresponding target genes were found, which included 9630 compound‐target corresponding connections. A total of 5542 NAFLD‐related targets were collected, resulting in 510 C&N target genes (contributing to 52.96% of CSP and 9.2% of NAFLD) and 641 corresponding compounds (contributing to 69.90% of CSP), which included 6877 target‐compound corresponding connections. At the C&N intersection, 25 NR targets were found (contributing to 2.7% of CSP, 0.45% of NAFLD and 52.08% of all NRs) and 311 corresponding compounds were found (33.91% of CSP and 51.64% of C&N), which included 895 target‐compound corresponding connections (Figures 1 and 9).

### 3.2 The components and targets of each CSP herb

**Figure 2:**
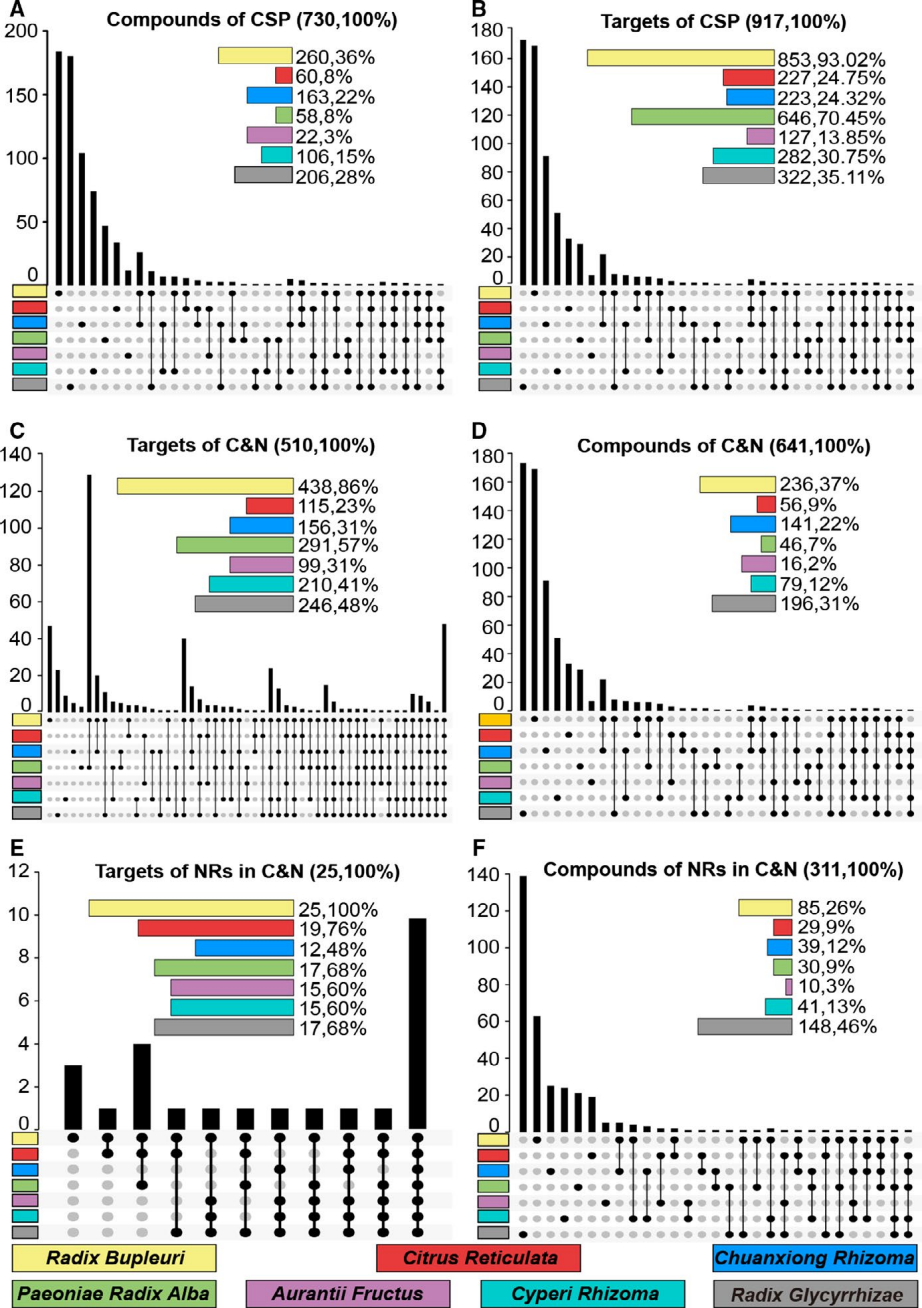
The amount of target‐compound interactions for each CSP herb. A, The CSP compound amount in each herb. B, The CSP targets amount in each herb. C, The C&N targets amount in each herb. D, The C&N compounds amount in each herb. E, The target amount of NRs among the C&N network in each herb. F, The compound amount of NRs among the C&N network in each herb. Above all the UpSet plots, the horizontal bars represent the total quantity and proportion of each herb, and the vertical bars represent the independent quantity of each herb and the quantities that intersect with that of the other herbs

**Figure 3:**
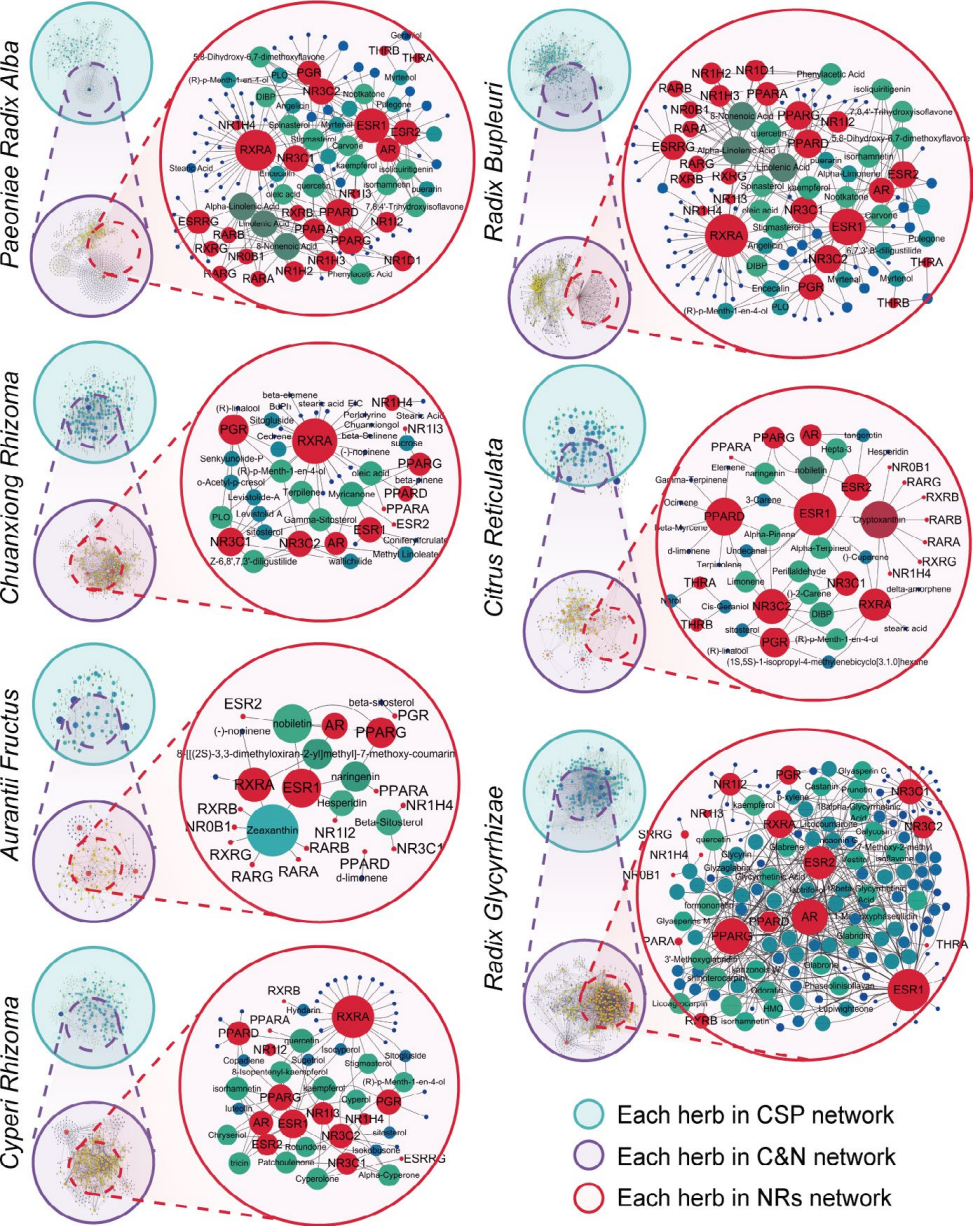
The NR target‐compound network of each herb in CSP. In the NR networks, the red nodes represent the targeted NRs, the blue and green nodes represent corresponding compounds, and the edges represent the connection between targets and compounds

Among the seven CSP herbs of *Radix Bupleuri*, *Citrus Reticulata*, *Chuanxiong Rhizoma*, *Paeoniae Radix Alba*, *Aurantii Fructus*, *Cyperi Rhizoma and Radix Glycyrrhizae*, 260, 60, 163, 58, 22, 106 and 208 compounds, respectively, were found, which corresponded to 853, 227, 223, 646, 127, 282 and 322 targets, respectively. In addition, 438, 115, 156, 291, 99, 210 and 246 C&N targets, respectively, were found, which corresponded to 236, 56, 141, 46, 16, 79 and 196 compounds, respectively. Finally, 25, 19, 12, 17, 15, 15 and 17 NR targets, respectively, were found among the C&N intersection, which correspond to 85, 29, 39, 30, 10, 41 and 148 compounds, respectively. *Radix Bupleuri* was the herb that contributed to the highest proportion of collected compounds and targets (Figures 2 and 3).

### 3.3 GO enrichment

**Figure 4:**
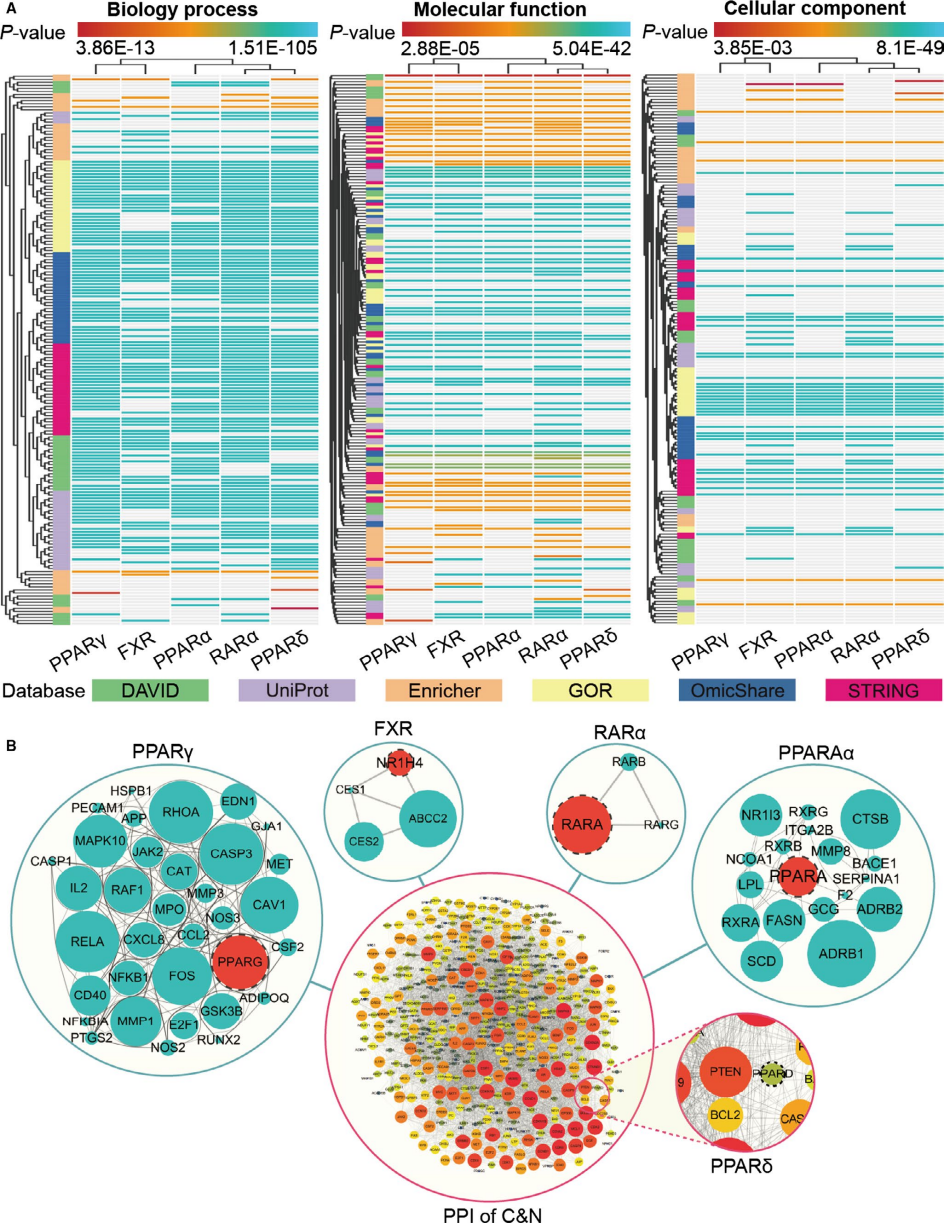
GO enrichment and PPI analysis. A, GO enrichment analysis of BP, MF and CC functions. All the target genes in the GO enrichment analysis were significant (*P* < .05). B, PPI analysis. The edge represents the connection between two proteins, and the size of the node represents the total number of connecting edges

A total of 510 C&N target genes were enriched in the BP, MF and CC analyses. Six database resources, DAVID, UniProt, Enrichr, GOR, OmicShare and STRING, were used for the integrated GO analysis, which resulted in 50,928 clusters in total and 540 clusters in the top 30 clusters determined in the BP, MF and CC analysis. After the NRs from the 540 clusters were ranked by frequency, the top 5 NRs were determined: peroxisome proliferator‐activated receptor gamma (PPARγ), farnesoid X‐activated receptor (FXR), peroxisome proliferator‐activated receptor alpha (PPARα), retinoic acid receptor alpha (RARα) and peroxisome proliferator‐activated receptor delta (PPARδ/β), and the frequencies were 250, 223, 221, 210 and 205, respectively (Figure 4).

### 3.4 PPI analysis

A total of 510 C&N target protein names were entered into the STRING database and a total of 6434 pairs of PPI connections were generated. PPARγ, PPARα, FXR, RARα and PPARδ had 83, 53, 40, 26 and 11 PPI connections, respectively. In total, 14 MCODE clusters were generated, and 4 MCODE clusters contained the top 5 NRs, which were PPARγ, PPARα, FXR and RARα, but no MCODE clusters contained PPARδ. Among the MCODE clusters, the cluster containing PPARγ had 43 targets, 247 interactions and a score of 14.970; the cluster containing PPARα had 18 targets, 36 interactions and a score of 4.235; the cluster containing FXR had 4 targets, 5 interactions and a score of 3.333; and the cluster containing RARα had 3 targets, 3 interactions and a score of 3.000 ( the cluster score represents the core density of the node and the topologically adjacent nodes; a higher score represents a more concentrated cluster), suggesting that the top 5 NRs may function through a multitarget synergistic mechanism with C&N targets (Figure 4).

### 3.5 Pathway enrichment analysis

**Figure 5:**
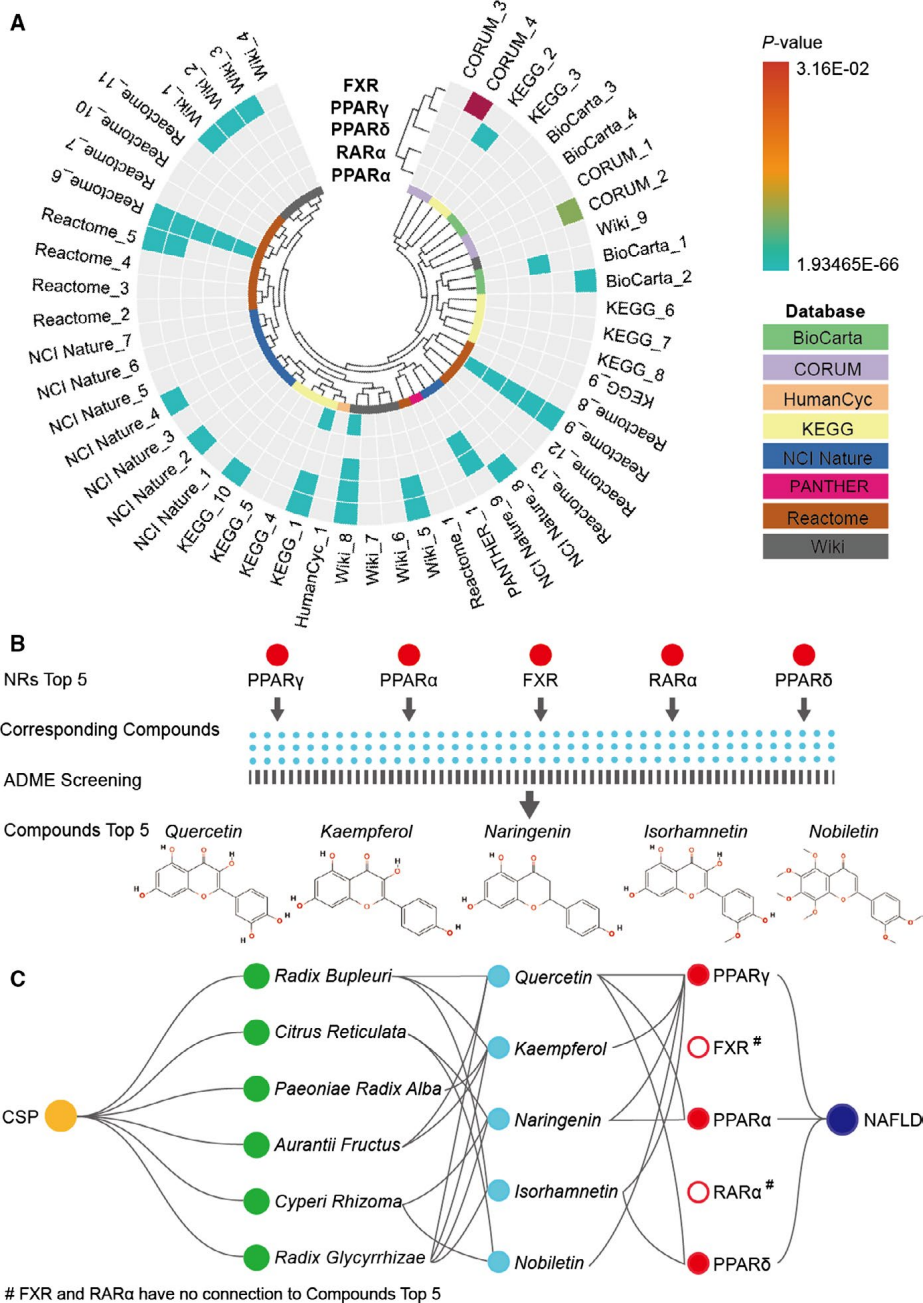
Pathway enrichment analysis of the NRs and the compounds and the screening workflow. A, Pathway enrichment analysis of the NRs. All the target genes in the paths were significant (*P* < .05). B, The workflow for screening the top 5 compounds. C, The relationship of CSP herbs‐top 5 compounds‐top 5 NRs‐NAFLD

Data from 8 database resources, including BioCarta, CORUM, HumanCyc, KEGG, NCI Nature, PANTHER, Reactome and Wiki, were integrated in the pathway enrichment analysis, which resulted in a total of 2791 paths, of which 210 paths were among the top 30 paths. Among these 210 paths, 51 paths had NRs, and 31 paths included the top 5 NRs. The paths containing the top 5 NRs included those related to the expression and regulation of nuclear receptor family proteins (NCI Nature_5 and 6, Reactome_6, Wiki_8, NCI Nature_3, BioCarta_1 and Reactome_11 and 12), NAFLD pathways (KEGG_9 and Wiki_7), metabolism of lipids, (Reactome_1, Reactome_3, Wiki_6, Reactome_10 and Reactome_13), peroxisome proliferation (BioCarta_1), biological oxidation or antioxidant generation (Reactome_2 and Wiki_9), tumours or cancers (KEGG_1 and 8), hepatitis B (KEGG_5), Th17 cell differentiation (KEGG_7), Toll‐like receptor pathway (BioCarta_3), CCKR signalling (HumanCyc_1 and Panther_1), calcineurin regulation in lymphocytes (NCI Nature_7), compound functionalization (Reactome_4), signal transduction (Reactome_5, 7 and 9) and cytochrome P450 (Reactome_7 and 8) (Figure 5).

### 3.6 Component screening

A total of 123 compounds corresponded to the top 5 NRs in total, of which PPARγ, PPARα, FXR, RARα and PPARδ corresponded to 92, 8, 9, 5 and 32, respectively. After the ADME screening based on OB and DL, only PPARγ, PPARα and PPARδ corresponded to the top 5 compounds. *Quercetin*, *kaempferol*, *naringenin*, *isorhamnetin* and *nobiletin* were screened as the top 5 compounds derived from six herbs: *Radix Bupleuri*, *Citrus Reticulata*, *Paeoniae Radix Alba, Aurantii Fructus*, *Cyperi Rhizoma* and *Radix Glycyrrhizae*, with each compound derived from at least two of these herbs, suggesting that the top 5 compounds may function through PPARγ, PPARα and PPARδ to affect NAFLD (Figure 5).

### 3.7 Rat general status

**Figure 6:**
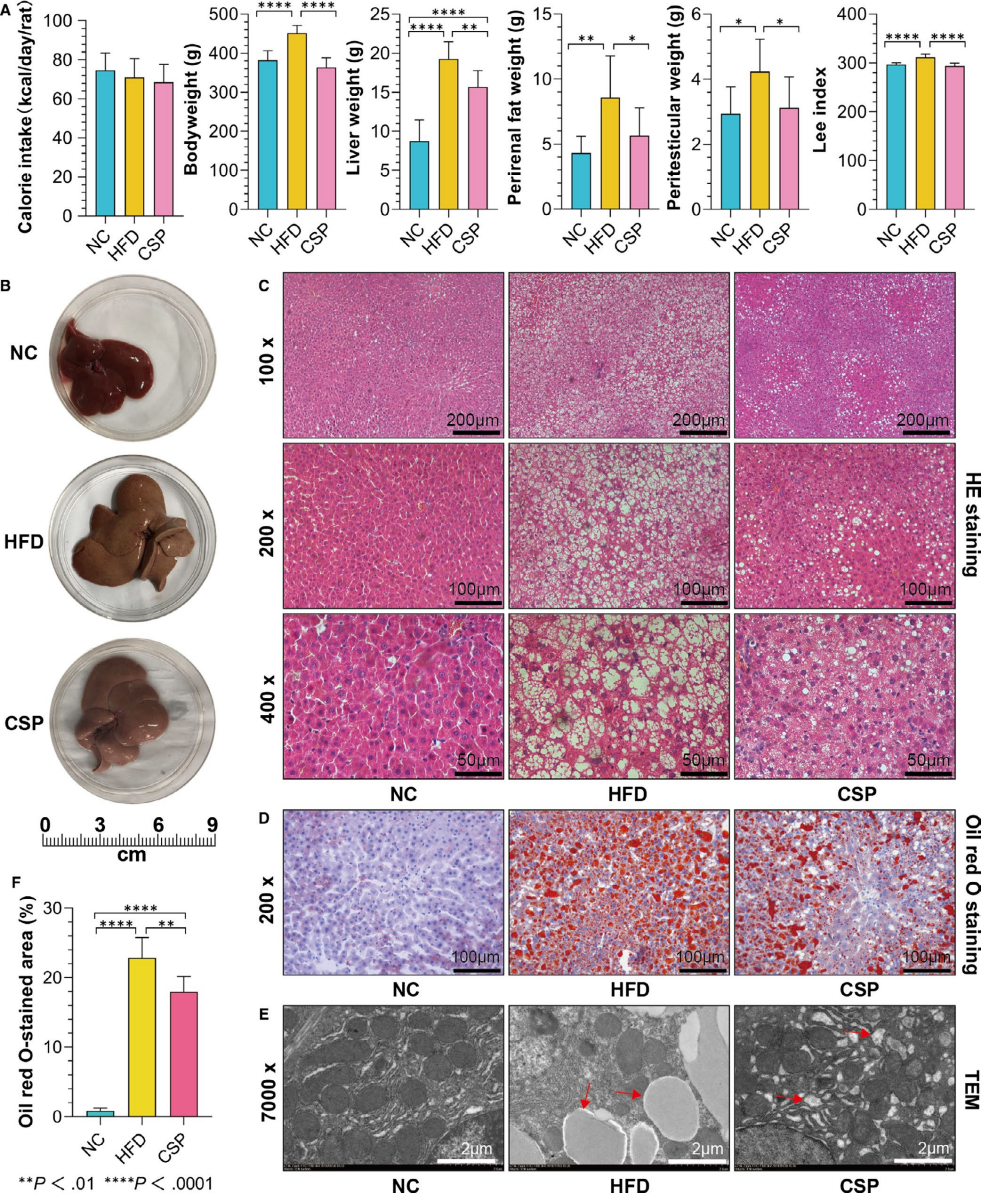
Rat general status and pathological observations. A, Rat general status. The bodyweight in the HFD group was higher than it was in the NC group (*P* < .0001) and the CSP group (*P* < .0001); the liver weight in the HFD group was higher than it was in the NC group (*P* < .0001) and CSP group (*P* = .0059), and it was lower for the NC group than it was for the CSP group (*P* < .0010); the perirenal fat weight in the HFD was higher than it was in the NC group (*P* = .0010) and CSP group (*P* = .0025); the peritesticular fat weight in the HFD group was higher than that it was in the NC group (*P* = .0116) and CSP group (*P* = .0309); and the Lee index of the HFD was higher than it was for the NC group (*P* < .0001) and CSP group (*P* < .0001) (n = 10). B, Morphology of the liver. C, HE‐stained liver tissue. D, Oil red O‐stained liver tissue. E, Transmission electron microscopy observation of the liver tissue. F, Oil red O‐stained area. The NC group had a lower lipid area than did the HFD group (*P* < .0001) and CSP group (*P* < .0001), and the lipid area for the CSP group was lower than it was for the HFD group (*P* = .0032) (n = 6)

The average daily calorie intake per rat indicated that the NC group rats ingested the most and the CSP group rats ingested the least, but the differences were not significant. The bodyweight and Lee index of the rats in the HFD group were significantly higher than those of the NC group and CSP group, and for the CSP group, the bodyweight and Lee index were slightly lower than those of the NC group, but the differences were not significant. The variable tendencies in liver weight, perirenal fat weight and peritesticular fat weight in rats were relatively similar among that rats; in the HFD group, these weights were significantly higher than they were in the NC or CSP group, and in the CSP group, they were higher than those in the NC group, but only the difference in liver weight was significant Figure 6.

### 3.8 Pathological observation of the liver

In general, the liver colour, blood flow, morphology, and the texture and tactility condition of liver tissues in the NC group were better than those in the HFD group or CSP group, whereas they were better in the CSP group than they were in the HFD group. The results of HE and Oil red O staining showed that the histological structure, cell morphology and cytoplasmic structures of the NC group were significantly more normal than those of the HFD and CSP groups, and only few lipid droplets were observed. In the HFD group, many deposited lipid droplets and an irregular cell morphology were observed. Compared with these parameters in the HFD group, the lipid droplet quantity was lower and the cell structure morphology was more regular in the CSP group. Under an electron microscope, for most of the mitochondria in the NC group, contours of the internal cristae were visible and a small amount of lipid was distributed in the cytoplasm. The HFD group had lipid droplets with a diameter of 2‐3 μm in the cytoplasm. The nuclei were concentrated; the mitochondria were diluted, swollen and distorted; the internal cristae were blurred; the endoplasmic reticulum quantity was reduced; necrotic hepatocytes and blurred structures were apparent; and the cell membrane had almost disappeared. In the CSP group, the lipid droplets were observed in hepatocytes, but the diameters of the lipids were mostly <1 μm. The mitochondria were swollen, but the internal cristae were still apparent, and the changes in the endoplasmic reticulum were significantly less dramatic than those in the HFD group. The lipid area calculation also showed that the NC group had significantly less lipid area than the HFD or CSP group, with that of the CSP group lower than that of the HFD group (Figure 6).

### 3.9 Biochemical parameters of the serum and liver homogenate

**Figure 7:**
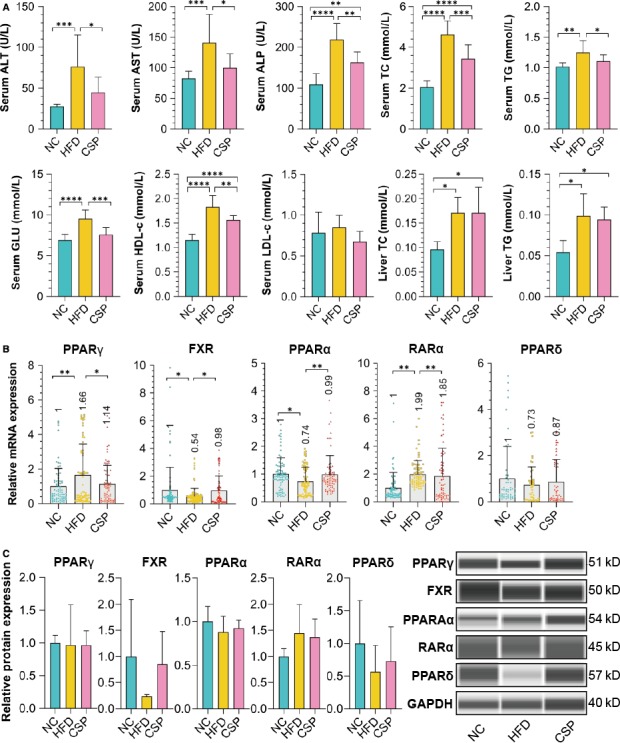
Biochemical measurements and the mRNA and protein expression of the top 5 NRs. A, The results of biochemical parameter measurements. Among the serum indicators, ALT in the HFD group was higher than it was in the NC group (*P* = .0004) and CSP group (*P* = .0222); the AST in the HFD group was higher than it was in the NC group (*P* = .0006) and CSP group (*P* = .0149); ALP in the HFD group was higher than it was in the NC group (*P* < .0001) and CSP group (*P* = .0013), and it was lower in the NC group than in the CSP group (*P* = .0017); in the HFD group, the TC level was higher than it was in the NC group (*P* < .0001) and CSP group (*P* = .0003), and in the NC group, it was lower than it was in the CSP group (*P* < .0001); in the HFD group, the TG level was higher than it was in the NC group (*P* = .0011) and CSP group (*P* = .0476); in the HFD group, the GLU level was higher than it was in the NC group (*P* < .0001) and CSP group (*P* = .0002); and in the HFD group, the HDL‐c level was higher than it was in the NC group (*P* < .0001) and CSP group (*P* = .0022), and in the NC group, it was lower than it was in the CSP group (*P* < .0001) (n = 10). Among the liver indicators, in the NC group, the TC level was lower than it was in the HFD group (*P* = .0276) and CSP group (*P* = .0427); in the NC group, the TG level was lower than it was in the HFD group (*P* = .0427) and CSP group (*P* = .0448) (n = 6). B, The mRNA expression of the top 5 NRs in the liver. PPARγ in the HFD group was expressed at a higher level than it was in the NC group (*P* = .0072) and CSP group (*P* = .0489); FXR in the HFD group was expressed at a lower level than it was in the NC group (*P* = .0222) and CSP group (*P* = .0331); PPARα in HFD group expressed lower than NC group (*P* = .0143) and CSP group (*P* = .0082); and RARα in the HFD group was expressed at a higher level than it was in the NC group (*P* = .0001) and CSP group (*P* = .0012) (n = 10). C, The expression of the top 5 NRs in the liver was measured by automatic Western blot analysis

In addition to LDL‐c, the other serum indicators were relatively consistent. The results of the NC and CSP groups indicated significantly lower levels than those in the HFD group, with some differences significant. According to the results from the liver homogenate, the TC and TG levels in the NC group were significantly lower than those in the HFD and CSP groups, whereas those in the HFD and CSP groups were relatively similar, with no significant differences (Figure 7).

### 3.10 The mRNA expression of the top 5 NRs in the liver

Compared with those in the NC group and CSP group, PPARγ and RARα were expressed at significantly higher levels in the HFD group with significance. FXR, PPARα and PPARδ were expressed at lower levels in the HFD group, but only the differences in FXR and PPARα were significant among groups. In contrast to those of the HFD group, the NRs in the CSP group exhibited a similar trend to that of the NC group, but there was no significant difference. These results indicated that PPARγ and RARα may have been down‐regulated in the NAFLD or high‐fat diet rat model, whereas FXR, PPARα and PPARδ may have been up‐regulated (Figure 7).

### 3.11 The protein expression of the top 5 NRs in the liver

FXR, PPARα and PPARδ were expressed at lower levels in the HFD group than in the NC group or CSP group, whereas the expression in the CSP group was more similar to that in the NC group than to that in the HFD group. The RARα expression level was higher in the HFD group and lower in the NC group and the CSP group. Generally, although no significance was found among the protein expression levels, the proteins were expressed following the approximate trend as the mRNA expression (Figure 7).

### 3.12 HPLC‐MS and molecular docking

**Figure 8:**
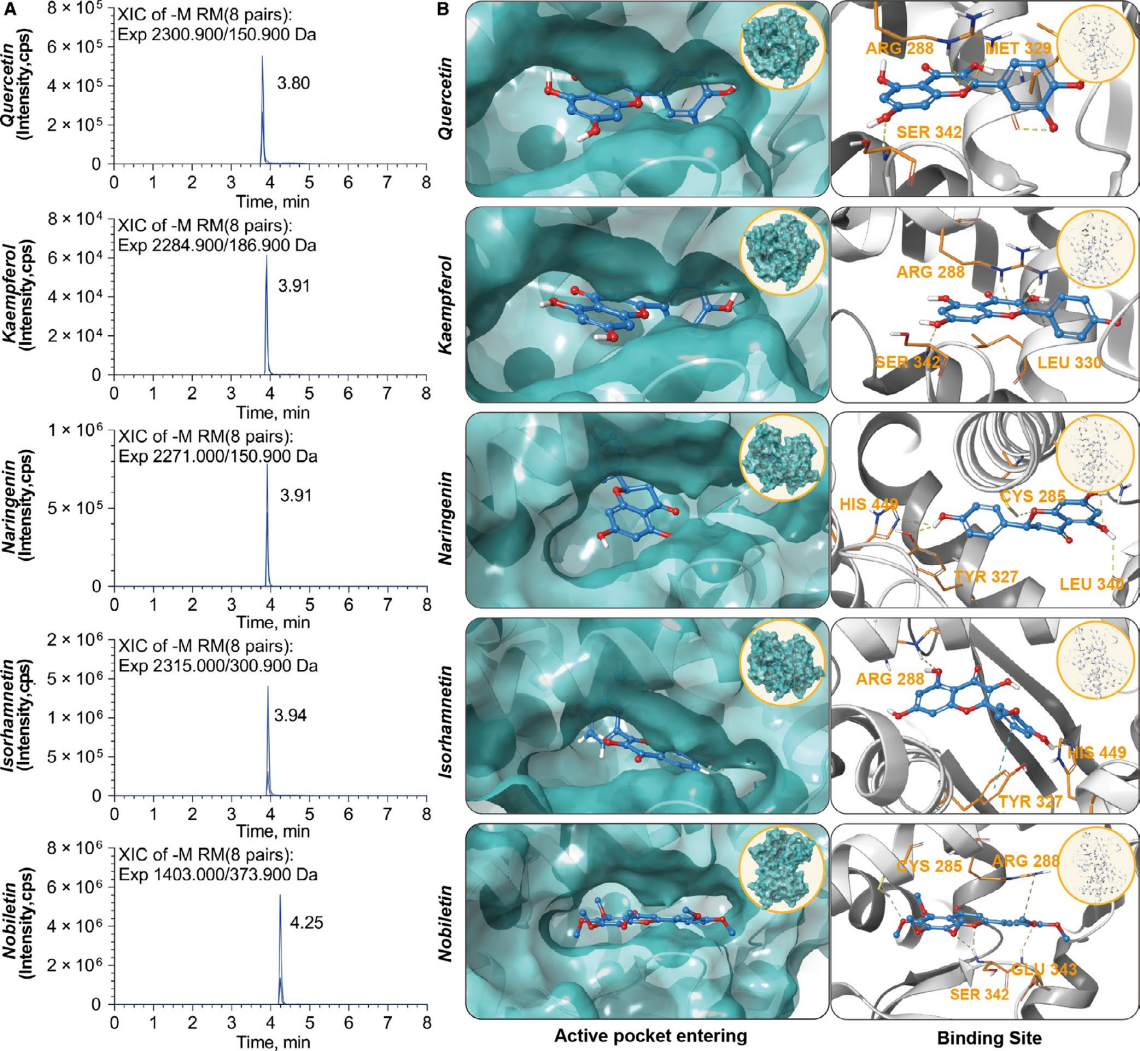
The results of HPLC‐MS and docking of the PPARγ molecules. A, The chromatogram of HPLC‐MS. B, Docking of PPARγ with the top 5 compounds. Quercetin interacts with 3 amino acids, ARG 288, MET 329 and SER 342, in the vicinity of the PPARγ active site, making them the main binding forces near the active site. Kaempferol interacts with 3 amino acids, ARG 288, LEU 330 and SER 342, near the PPARγ active site and forms 4 hydrogen bonds, thus promoting binding. Naringenin formed a network of five hydrogen bonds with PPARγ through 4 amino acids, CYS 285, HIS 440, TYR 327 and LEU 340, near the active site, establishing stable binding. The phenolic hydroxyl in isorhamnetin interacted through 2 hydrogen bonds at ARG 288 and HIS 449 in PPARγ, thus forming a Pi‐Pi interaction with TYR 327. Nobiletin formed 4 hydrogen bonds with 3 amino acids, CYS 285, SER 342 and GLU 343, in PPARγ and formed a Pi‐n interaction between a guanidine and ARG288

According to the chromatogram obtained by HPLC‐MS, the peaks representing *quercetin*, *kaempferol*, *naringenin*, *isorhamnetin* and *nobiletin* were detected, and the peak out‐flow times were 3.80, 3.91, 3.91, 3.91, 3.94 and 4.25 minutes, respectively; the peak intensities were 5.54*e*5, 6.14*e*44, 5.54*e*5, 1.40*e*66 and 5.60*e*6 cps, respectively. Because only PPARγ, PPARα and PPARδ were associated with the top 5 compounds, the protein numbers of PPARγ‐5F9B, PPARα‐4CI4 and PPARδ‐3TKM in the PDB database were finally selected for further analysis, and PPARγ is presented as an example of the docking process. Each small molecule could enter into the active pocket of the protein and showed proper matching features, making it possible for the small molecules to bind with the active site. There were still some cavities near the small molecule, which made it possible for further structural transformation of the small molecules. Furthermore, all the docking processes had good scores (Figure 8) (Table 1).

**Table 1:** The score of molecular docking

Docking compounds and proteins	PPARγ	PPARα	PPARδ
Quercetin	−7.9	−7.8	−8.4
Kaempferol	−7.6		
Naringenin	−7.7	−7.7	
Isorhamnetin	−7.9		−8.2
Nobiletin	−7.1		

## 4 DISCUSSION

### 4.1 Network pharmacology and CSP

**Figure 9:**
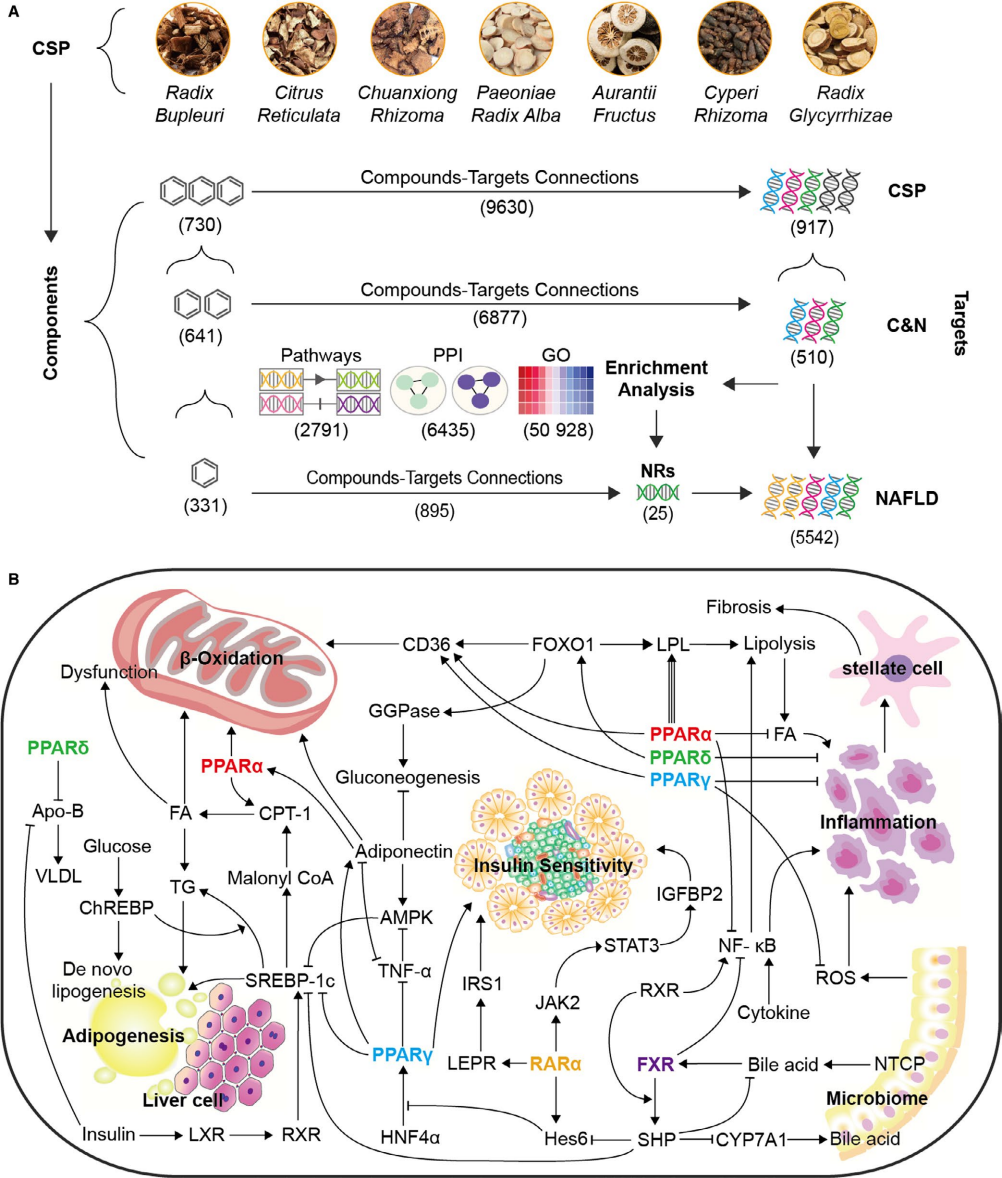
The complex pharmacological processes of CSP and the top 5 NRs in NAFLD. A, The complex compound‐target network of CSP herbs in NAFLD. B, Summary of the mechanisms of the top 5 NRs in NAFLD. Some key factors in various processes, including insulin sensitivity, oxidative stress, adipogenesis, inflammation and microbiome, are shown

The network pharmacology method is essentially a combination of clustering algorithms and network topology. As the complex data interaction relationship is well presented by the visualized node interactions, this method is often used to analyse TCM herbs.^11^, ^12^, ^13^, ^14^ Based on the 730 compounds and 917 corresponding target genes found, CSP has a large quantity of chemical components and corresponding targets. If the compounds and the targets only had one‐to‐one relationships, then they would form nearly a thousand pairs of interacting connections. It is widely known that a target generally functions through several synergistic mechanisms with the related targets and that there may be many interactions between the targets. Therefore, it was not surprising that 50 928 GO enrichment clusters, 6435 pairs of PPI connections and 2791 pathways were found. In addition, most of the CSP compounds have pharmacological functions through multiple NAFLD targets. Therefore, the whole therapeutic process from compounds to targets and finally to NAFLD was composed of a very large and complex hierarchical crossover compound‐target network (Figure 9). With the current technology level, verification of the whole potential target network is difficult, but pinpointing the factors with high research potential is possible. For example, significant differences could be used as a screening condition after enrichment to determine the clusters that were more significant. Because of the large number of screened targets, the biological mechanism covered almost all aspects of the development of NAFLD, and biological functions could also be used as screening conditions, which contribute to a more precise focus on the highly significant targets with specific biochemical functions.

### 4.2 The effects of CSP on NAFLD

The development of NAFLD includes a wide pathological process of mechanisms involving insulin resistance, oxidative stress, endoplasmic reticulum stress, apoptosis, lipotoxicity, inflammation and the microbiome.^15^ CSP is widely used in China and other East Asian countries, and the treatment spectrum involves NAFLD,^8^ NASH,^9^ functional dyspepsia,^16^ depression,^17^ atherosclerosis,^18^ etc, although the specific components and targets of CSP that are most important in NAFLD are currently unclear. All the components and targets of CSP revealed in this study seem to cover most of the biological mechanisms of NAFLD,therefore, we inferred that CSP has a broad impact on many factors closely related to NAFLD. Obesity and lipid accumulation are important cofactors in the development of NAFLD.^15^, ^19^, ^20^ The alleviation of overweight and lipid accumulation of the liver, perirenal and peritesticular tissue were apparently found in the CSP when compared to those in the HFD group. According to the pathological observations, fewer lipid droplets were found in the CSP group than in the HFD group. The pathological results also showed that the lipid area of the HFD group was 22.86%, while that of the CSP group was only 17.96%, which was 4.9% lower. According to relevant reports, the criterion of at least 5% of the hepatocytes showing steatosis, as indicated by large cysts, large pure droplets or mixtures of small droplets and large droplets, can be used as the basis for an NAFLD diagnosis.^21^ Therefore, in our study, the NAFLD rat model was successfully established, with obviously better liver adipose pathology amelioration in the CSP group. In addition, in the HFD group and CSP group, no obvious inflammations were observed in the liver tissue sections, which indicated that the model was simple fatty liver without a sign of NASH. The biochemical results indicated that the AST, ALT and ALP levels in the CSP group were significantly lower than those in the HFD group. Currently, no recognized biochemical diagnostic indicators are available for NAFLD, AST, ALT and ALP can only be used only as the auxiliary diagnostic indicators for NAFLD or for differential diagnosis with NASH and fibrosis.^3^, ^22^, ^23^ Abnormal blood lipids are characterized by an increase in TG and LDL‐C, and a decrease in HDL‐C levels.^3^, ^24^, ^25^ GLU is also an important biochemical factor in NAFLD and an important substrate in the synthesis of lipids.^26^, ^27^ In the present study, the serum levels of TG, TC, HDL‐C and GLU in the CSP group were significantly lower than those in the HFD group. The biochemical and pathological results indicate that CSP has the prospect of lowering bodyweight and liver and blood lipids in NAFLD patients.

According to the mRNA and protein expression analysis, the top 5 NRs exhibited different expression levels in the different groups. The HFD group largely exhibited the opposite trend as that shown by the NC group and CSP group. The CSP group exhibited a trend similar to that of the NC group, according to the qRT‐PCR results based on a relatively large sample size, with approximately eighty in each group, although not all the differences were significant, especially in terms of protein expression. PPARα, PPARδ and PPARγ are peroxisome proliferator‐activated receptors (PPARs), which are important factors in the development of NAFLD.^28^, ^29^ PPARα is primarily expressed in the liver and up‐regulated in fatty liver, which can accelerate the formation of FAs caused through the lipolysis in liver adipose tissue by regulating the expression of apolipoproteins. PPARα also increases the HDL‐c level and decreases the LDL‐c level in blood, and reduces the blood glycerine level by directly up‐regulating the expression of genes involved in glycogenesis from glycerol.^28^, ^29^, ^30^, ^31^ PPARδ is primarily expressed in skeletal muscle and down‐regulated in fatty liver. Activation of PPARδ can enhance FA transport and oxidation, and enhance glucose homeostasis by increasing insulin sensitivity and inhibiting glucose output. It also weakens the macrophage inflammatory response and increases the blood HDL‐c level.^28^, ^29^, ^32^, ^33^ PPARγ is primarily expressed in adipose tissue and up‐regulated in fatty liver, where it can control adipocyte differentiation. However, PPARγ can also increase insulin sensitivity, promote FA absorption by adipocytes and ultimately reduce FA delivery to the liver.^28^, ^29^, ^34^ FXR can maintain bile acid homeostasis and induce acute phase reactive proteins that play important roles in the regulation of lipid metabolism and inhibition of liver inflammation and are generally underexpressed in the liver of NAFLD patients.^35^, ^36^ It has been reported that RARα can form a new transcription cascade with PPARγ and reduce the accumulation of TG in the liver^37^ (Figure 9).

Among the components screened, interestingly, the top 5 were flavonoids with similar chemical structures. *Quercetin* can reduce the accumulation of triglycerides, decrease insulin resistance and inhibit inflammatory cytokine secretion, increasing the antioxidative capacity of cells, and even ameliorate NAFLD by reestablishing a balance in the intestinal flora.^38^, ^39^ *Kaempferol* can activate PPARα and PPARδ through protein kinase B to reduce the accumulation of TGs in the liver,it can combine with PPARα to stimulate FA oxidation signalling that inhibits the accumulation of adipogenic transcription factors and lipids in adipocytes.^40^, ^41^ *Naringin* can reduce the biomarker levels of lipid peroxidation and protein carbonylation, increase the defensive ability of antioxidants, clear the active oxygen and regulate the signal pathways related to FA metabolism, making cells conducive to FA oxidation and reduced lipid accumulation.^42^ *Isorhamnetin* can inhibit the differentiation of adipocytes that is induced by rosiglitazone, a PPARγ agonist, and reduce the development of obesity and the fatty degeneration of the liver caused by a high‐fat diet and leptin deficiency.^43^ *Nobiletin* can prevent the inflammation, insulin resistance, dyslipidaemia and fatty liver caused by a high‐fat diet.^44^ According to their functions, the top 5 compounds may pharmacologically act on NAFLD and may function through PPARs. Although these compounds can bind with PPARα, PPARδ and PPARγ well, we cannot overlook the fact that the screened compounds and target genes are involved in a small part of CSP biofunctions. Other compounds and targets have potential and should be further studied.

## 5 CONCLUSION AND FURTHER RESEARCH

In this study, CSP showed positive effects on some factors closely related to NAFLD, such as bodyweight and lipid accumulation. The network pharmacology approach with multiple databases, as adopted in this study, was useful in screening potential targets, and the screened compounds and genes found exhibited therapeutic potential for NAFLD. The final five compounds that were screened out are flavonoids, which can combine with the screened‐out target genes PPARγ, PPARα and PPARδ, and thus show potential as drugs for NAFLD. This study also provided a methodological exploration based on network pharmacology. Although we tried to rescreen the C&N targets through enrichment analysis, the target frequency presented in clusters, and ADME screening, the biological function classification was still necessary to identify targets precisely. In vivo and in vitro experiments need to be conducted in the next step to investigate the specific pharmacological effect of the top 5 compounds on NAFLD. In addition, various high‐throughput screening methods, such as sequencing and genomics or proteomics analyses, can be combined with target screening to produce more solid evidence in support of these screening results.

## CONFLICT OF INTEREST

The authors declared that they have no conflicts of interest related to this study.

## AUTHOR CONTRIBUTIONS

Yupei Zhang and Qinhe Yang designed the study. Huan Nie and Yuanjun Deng conducted the network pharmacology analysis and verification experiments. Chuiyang Zheng, Maoxing Pan and Jiqian Xie collected data. Huan Nie wrote the manuscript. Huan Nie designed the figures, and Jiqian Xie revised the manuscript. Yupei Zhang provided suggestion for revising the manuscript. All authors read and approved the final manuscript.

## Supporting information

Table S1

Table S2

## Data Availability

The data used to support the findings of this study are available from the corresponding author upon reasonable request.
