# Supplementary material for: A network pharmacology‐based approach to explore the effects of Chaihu Shugan powder on a non‐alcoholic fatty liver rat model through nuclear receptors
Source: J Cell Mol Med. 2020 Mar 18;24(9):5168–84. doi: 10.1111/jcmm.15166 (PMC7205817; doi:10.1111/jcmm.15166)
Supplement: Supplementary file 1 — Table S1 [file JCMM-24-5168-s001.docx]

Table S1. Databases used in target screening

| Database | Inroduction | Function |
| --- | --- | --- |
| TCMSP | A pharmacology platform captures the relationships between drugs, targets and diseases. (lsp. nwu. edu. cn) | Herbal ingredients and targets collection |
| BATMAN-TCM | The first online bioinformatics analysis tool for molecular mechanism research of TCM. (http://bionet. ncpsb. org/batman-tcm/) |  |
| TCMID | A traditional Chinese medicine integrative database for herb molecular mechanism analysis. (www. megabionet. org/tcmid) |  |
| TCMGeneDIT | A database mined from biomedical literature providing association information about genes, diseases, TCM effects and TCM ingredients. (http://tcm. lifescience. ntu. edu. tw) |  |
| DrugBank | A bioinformatics and cheminformatics resource that combines detailed drug data with comprehensive drug target information. (www. drugbank. ca) | Molecular information and targets collection |
| PubChem | A chemistry molecules database that contain 96 million compounds. (pubchem. ncbi. nlm. nih. gov) |  |
| NCBI | American national center for biotechnology information. (www. ncbi. nlm. nih. gov) | Disease targets collection |
| GeneCards | A integrative database providing comprehensive information on all annotated and predicted human genes. (www. genecards. org) |  |
| DisGeNET | A discovery platform containing one of the largest publicly available collections of genes and variants associated to human diseases. (www. disgenet. org) |  |
| UniProt | A comprehensive resource for protein sequence and annotation data. (www. uniprot. org) | Protein/Gene annotation |
| ENSEMBL | One of the largest genome annotation webs. (www. ensembl. org) | Protein-gene name conversion |
| DAVID | An onlion database for annotation, visualization and integrated discovery. (david. ncifcrf. gov) | Gene ontology enrichment and pathway enrichment analysis |
| Enrichr | A comprehensive gene set enrichment analysis web server |  |
| Gene Ontology Resource(GOR) | The world’s largest source of information on the functions of genes. |  |
| OmicShare | An open platform for researchers to share knowledge and analyze biological information. (www. omicshare. com) |  |
| BioCarta | A sets of proteins participating in pathways. (www. biocarta. com) |  |
| CORUM | The comprehensive resource of mammalian protein complexes. (mips. helmholtz-muenchen. de/corum/) |  |
| HumanCyc | A manually curated database of enzymatic reactions and metabolic pathways. (humancyc. org/) |  |
| KEGG | A database resource of functions and utilities of the biological system, such as the cell, the organism and the ecosystem, from molecular-level information. (www. genome. jp/kegg/) |  |
| NCI Nature | A collection of curated and peer-reviewed signaling pathways composed of human biomolecular interactions and cellular processes. (pid. nci. nih. gov) |  |
| PANTHER | A classification system designed to classify proteins and their genes in order to facilitate high-throughput analysis. (http://www. pantherdb. org/pathway/) |  |
| Reactome | A pathway database providing intuitive bioinformatics tools for the visualisation, interpretation and analysis of pathway knowledge. (reactome. org) |  |
| Wiki Pathways | A database of biological pathways maintained by and for the scientific community. (www. wikipathways. org) |  |
| STRING | A database of known and predicted protein-protein interactions. (string-db. org) | Protein-protein interaction |
| PDB | A resource of the 3D shapes of proteins, nucleic acids, and complex assemblies(www. rcsb. org) | Molecular docking |
