# Supplementary material for: A network pharmacology‐based approach to explore the effects of Chaihu Shugan powder on a non‐alcoholic fatty liver rat model through nuclear receptors
Source: J Cell Mol Med. 2020 Mar 18;24(9):5168–84. doi: 10.1111/jcmm.15166 (PMC7205817; doi:10.1111/jcmm.15166)
Supplement: Supplementary file 2 — Table S2 [file JCMM-24-5168-s002.docx]

Table S2. Primer information

| Gene | Primer Sequence | | Amplified  Length（bp） |
| --- | --- | --- | --- |
|  | Forward primer （5' - >3'） | Reverse primer （5' - >3'） |  |
| PPARγ | GAGGCCAGCATGGTGTAG | TTCAAGGGTGCCAGTTTC | 151 |
| PPARα | TTCCTGCGAGTATGACCC | AAAACTGAAGGCAGAAATCC | 149 |
| NR1H4 | ACCCAGGTTGGAATAATAGG | ACAAGCCACGGACGAGTT | 175 |
| RARα | TACTCCGAAGGTCCGTGA | ACCGACTTGGTCTTTGCC | 244 |
| PPARδ | GGACTGGCAGCGGTAGAACAC | CACCCTTCATCATCCACGACA | 128 |
